# The Enhanced Light Absorptance and Device Application of Nanostructured Black Silicon Fabricated by Metal-assisted Chemical Etching

**DOI:** 10.1186/s11671-016-1528-0

**Published:** 2016-07-02

**Authors:** Hao Zhong, Anran Guo, Guohui Guo, Wei Li, Yadong Jiang

**Affiliations:** State Key Laboratory of Electronic Thin Films and Integrated Devices, University of Electronic Science and Technology of China, Chengdu, 610054 China; School of Optoelectronic Information, University of Electronic Science and Technology of China, Chengdu, 610054 China

**Keywords:** MCE, Black silicon, Photoelectronic detector, Device responsivity, Absorptance

## Abstract

We use metal-assisted chemical etching (MCE) method to fabricate nanostructured black silicon on the surface of C-Si. The Si-PIN photoelectronic detector based on this type of black silicon shows excellent device performance with a responsivity of 0.57 A/W at 1060 nm. Silicon nanocone arrays can be created using MCE treatment. These modified surfaces show higher light absorptance in the near-infrared range (800 to 2500 nm) compared to that of C-Si with polished surfaces, and the variations in the absorption spectra of the nanostructured black silicon with different etching processes are obtained. The maximum light absorptance increases significantly up to 95 % in the wavelength range of 400 to 2500 nm. Our recent novel results clearly indicate that nanostructured black silicon made by MCE has potential application in near-infrared photoelectronic detectors.

## Background

In recent years, several approaches have been explored to fabricate micro- and nanostructures on polished surfaces of monocrystalline silicon, aiming to reduce light reflectance in photovoltaic devices and photoelectronic detectors [1–3]. Microstructured black silicon obtained by femtosecond laser pulses in SF_6_ environment is one of the most attractive materials in these application fields of photoelectronic devices [4–6]. As a result, such black silicon displays strong light absorptance in the wavelength range of 250 to 2500 nm due to the impurity band gap levels induced by the doped chalcogen [7, 8]. However, the technology of black silicon made by femtosecond laser is a high-cost and time-consuming process.

Many micro- and nanostructured black silicon materials can also be manufactured by using metal-assisted chemical etching (MCE) treatment [9–12]. In the MCE process by using Ag particles as catalyst, a chemical reduction reaction of Ag^+^ will happen on the surface of silicon substrate, and at the same time, the silicon atoms around Ag particles are oxidized and dissolved, generating pores or wires and finally forming a layer called black silicon on the top of the substrates [13]. Generally, HF/AgNO_3_ mixed solution or Tollens’ reagent is widely used to deposit Ag particles. In addition, a novel Ag deposition method by fabricating 2D non-close-packed silica colloidal crystal on silicon surface is also reported, by which ordered silicon nanowire arrays can be fabricated via nanosphere lithography and MCE process [14]. The specific geometries of the corroded silicon structures depend mainly on the initial distribution of Ag particles and etching duration. Under certain conditions, aligned silicon nanocone arrays can be obtained [15].

As a new functional material, black silicon has drawn worldwide attention in recent years. It is an ideal material used for sensitive photoelectronic detectors [16–18], solar cells, biochemical sensors [19, 20], display devices [21, 22], and optical communication objects [23]. Nanostructures of black silicon have been the focus of intense researches in recent years due to their extensive device application and possibility of investigation on 1/f noise [24, 25]. Therefore, the nanostructured black silicon created by MCE method appears to be an ideal material for photoelectronic detectors because of its outstanding light management properties in the spectral range of 400 to 2500 nm. In addition to enhancing efficiency, black silicon can provide significant savings in manufacturing costs as there is no need to deposit a separate antireflection coating [26]. Different from MSM structures, PIN structures have wide depletion layer so that it can reduce the influence of carrier diffusion movement to achieve the purpose of improving response speed. PIN photoelectronic detectors have the potential to develop high-speed optoelectronic integrated circuit (OEIC) due to their short response time, low dark current, and high sensitivity.

In this article, we report the light absorptance enhancement of nanostructured black silicon fabricated by MCE method and its application in Si-PIN photoelectronic detector. The effect of different structured morphologies on the light absorptance in the wavelength range of 400 to 2500 nm have been studied, and the detector based on nanostructured black silicon has been extensively investigated with an emphasis on device responsivity at 1060 nm.

## Methods

N-type silicon wafers with a thickness of 500 μm and a resistivity of 2500 to 3000 Ω cm were used. Silver mirror reaction was applied to deposit Ag particles. Silicon pieces (15 × 15 cm^2^) cut from silicon wafer were first immersed into Tollens’ reagent prepared by 0.04 mol/L AgNO_3_, 2 % NaOH, 2 % ammonia (NH_3_ · H_2_O) and 1.5 % glucose at room temperature. Different samples were obtained in our experiment by varying the silvering duration to be 40, 60, and 80 s, respectively. In order to investigate the effect of etching period on light absorptance of black silicon, we moved the optimized samples (Ag deposition for 60 s) into an etching solution, which was made of 10 % HF and 0.6 % H_2_O_2_, for 15, 45, and 60 min, respectively. When the etching processes were over, the silicon pieces were dipped into an aqueous solution of HNO_3_ and then rinsed with deionized water to remove any residual Ag.

**Fig. 1:**
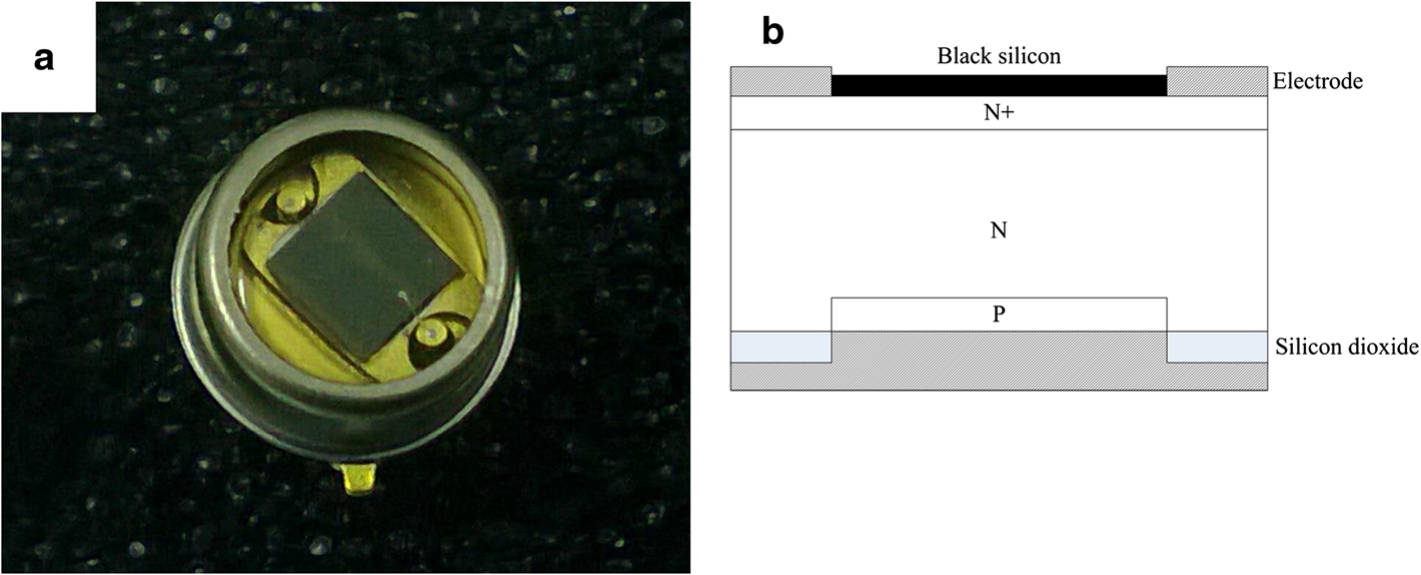
Real image of the detector (**a**) and structure (**b**) of Si-PIN detector based on nanostructured black silicon

Figure 1 shows the real device image (a) and structure of Si-PIN photoelectronic detector with nanostructured black silicon formed on the front surface (b), respectively. First, a p-type layer was fabricated by diffusing into a lightly doped n-type wafer on polished surface. Second, the wafer was grinded to a thickness of about 300 μm on the back side of the wafer. Third, a P-doped N^+^ layer was made on the grinded surface of the wafer, and then the nanostructured black silicon was formed on the top of N^+^ layer. Finally, ohmic contact was performed by the deposition of metal electrodes on both sides of the wafer. The photosensitive surface is square in shape with a side dimension of 3.6 mm (shown as in Fig. 1a).

The morphologies of black silicon and distribution of Ag particles were characterized by a field emission scanning electron microscope (SEM, JSM-7500F). The light absorptance was obtained at room temperature using a fiber optic spectrometer (NIR2500) equipped with an integrating sphere (Idea Optics, IS-20-5). The detector responsivity was measured by using an optical power meter (OPHIR, Vega), an optical chopper (Scitec Instruments, Model-300CD), and a Keithley 2400 apparatus under the dark room environment. In order to ensure the accuracy of the measurement, we carried out calibration before test and each of these measurements was performed on a few samples (usually 4 to 6).

## Results and Discussion

**Fig. 2:**
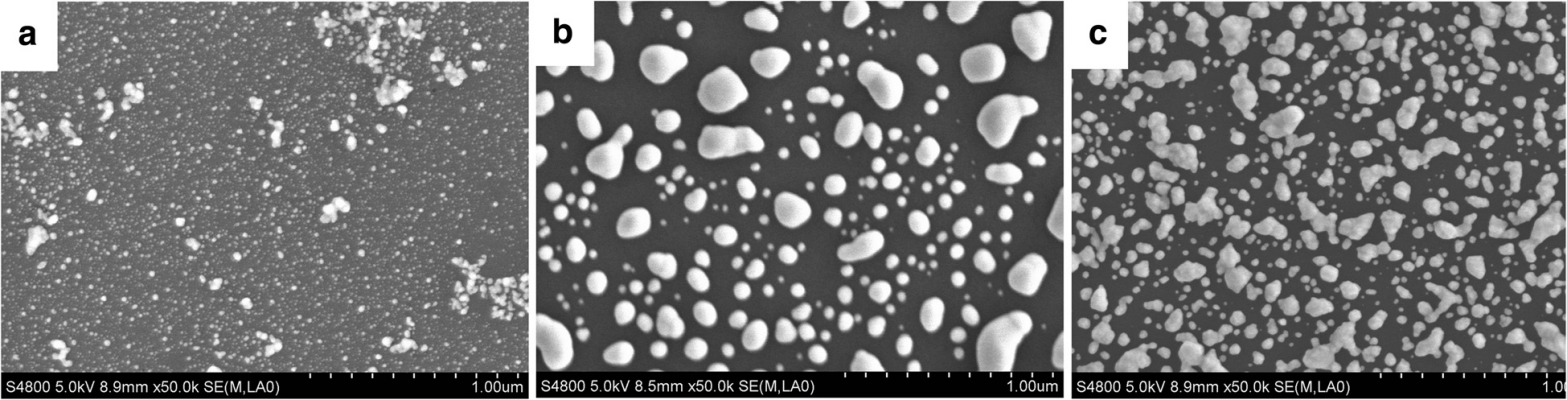
The deposited Ag particles on silicon surface at different silvering time: **a** 40 s, **b** 60 s, and **c** 80 s

Figure 2 shows the typical SEM image of Ag particles deposited on the silicon substrate for three different silvering time. The equation of silver mirror reaction used to deposit Ag particles as follows:

1 $$ {C}_6{H}_{12}{O}_6+2\left[Ag{\left(N{H}_3\right)}_2\right]OH\to {C}_5{H}_{11}{O}_5 COON{H}_4+3N{H}_3+2Ag\downarrow +{H}_2O $$

It is clearly shown that the Ag particles in Fig. 2a distribute sparse and only a few deposit on the silicon surface. As shown in Fig. 2c, the over-reaction leads to a state where Ag particles have adhered together. Figure 2b, however, presents uniformly distributed particles covering more than half areas of the silicon surface and leaving appropriate gaps between particles. It can be seen from the SEM images that there will be more narrow space between two particles as the silvering time increases.

During the second step of MCE, these Ag particles will gradually migrate into silicon substrate while nanometer-sized cones protrude outwardly from the space between Ag particles. The main chemical reaction occurs in the etching process can be described as follows:

2 $$ Si+{H}_2{O}_2+6HF\to 2{H}_2O+{H}_2Si{F}_6+{H}_2\uparrow $$

**Fig. 3:**
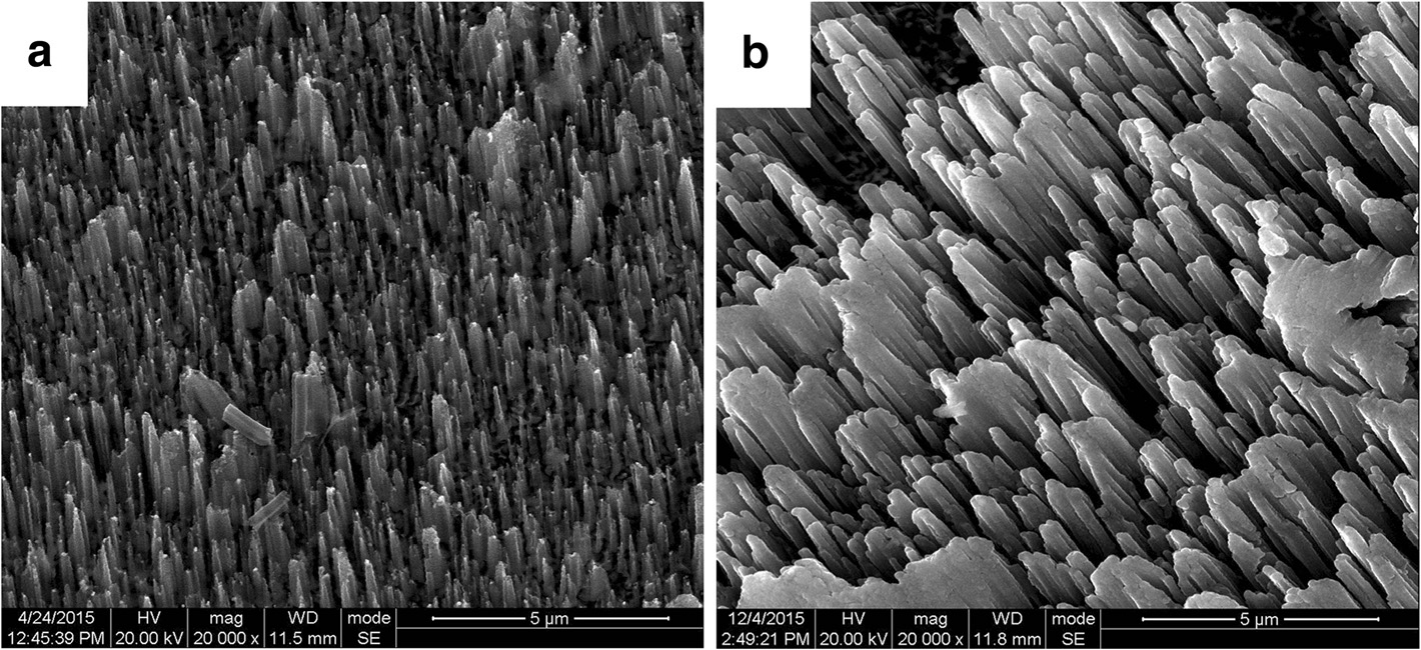
Two typical SEM images of silicon nanocone arrays made by MCE for different etching time: **a** 15 min and **b** 60 min

As a result, the etched surface of the silicon substrate will be covered with aligned silicon nanocone arrays which are perpendicular to the surface of substrate (as in Fig. 3). It can be seen from Fig. 3b that the sample etched for 60 min shows the best aspect ratio, in which the average diameter and length of silicon nanocone arrays are about 100 nm and 2.5 μm, respectively. Obviously, the morphology of these silicon nanocone arrays can be well controlled by varying Ag deposition and etching duration.

**Fig. 4:**
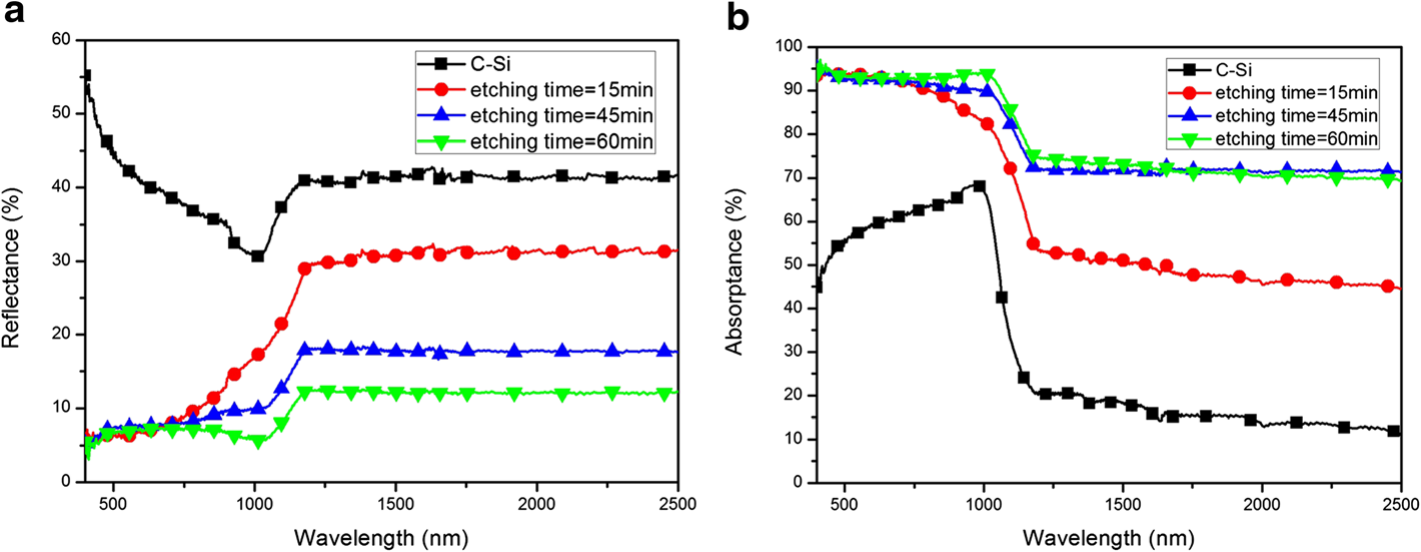
Reflectance (**a**) and absorptance (**b**) of C-Si and black silicon fabricated for different etching time

As shown in Fig. 4a, the light reflectance is obviously suppressed due to the silicon nanocone arrays existing on the surface of the samples. The reflectance of ordinary C-Si is much higher than that of the specific C-Si with nanostructured surface. It can be easily understood that the longer the etching time, the higher the silicon nanocone arrays. This is the reason why the reflectance of three kinds of samples is different.

Figure 4b represents the light absorptance of samples with nanostructured networks fabricated on the surface of the C-Si at different etching time. It clearly shows that C-Si with etched black silicon arrays, compared to ordinary C-Si, remarkably enhances light absorptance throughout the wavelength range of 400 to 2500 nm. The sample etched for 60 min presents the highest absorptance, up to 95 % in the NIR range (800 to 2500 nm), and the average absorptance of this sample reaches 91 % in the wavelength range of 400 to 2500 nm. This high absorptance mainly comes from the multiple reflection of light among silicon nanocone arrays, increasing the light path and the capture ratio of photon. From the absorption spectra illustrated in Fig. 4b, it can be easily found that the curves of samples with nanostructured networks show an obvious increase in the near-infrared band. This absorptance enhancement in the wavelength range of 400 to 2500 nm can be attributed to the increase in reflection times taken among the nanostructured silicon arrays.

**Fig. 5:**
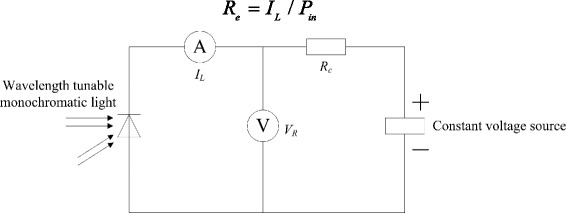
The principle diagram of the detector responsivity measurement

Figure 5 shows the principle diagram of detector responsivity measurement used in this paper. In order to ensure the accuracy of the measurement, all the instruments must be calibrated before testing. And then, each value of light power *P*_in_ illuminating the detector photosensitive area at different specific wavelength can be obtained. During testing, a photocurrent *I*_*L*_ can be recorded when the detector is applied to a reverse bias about 12 V as in Fig. 5. At last, the detector responsivity *R*_*e*_ at each specific wavelength can be calculated by formula (3).

3 $$ {R}_e={I}_L/{P}_{\mathrm{in}} $$

**Fig. 6:**
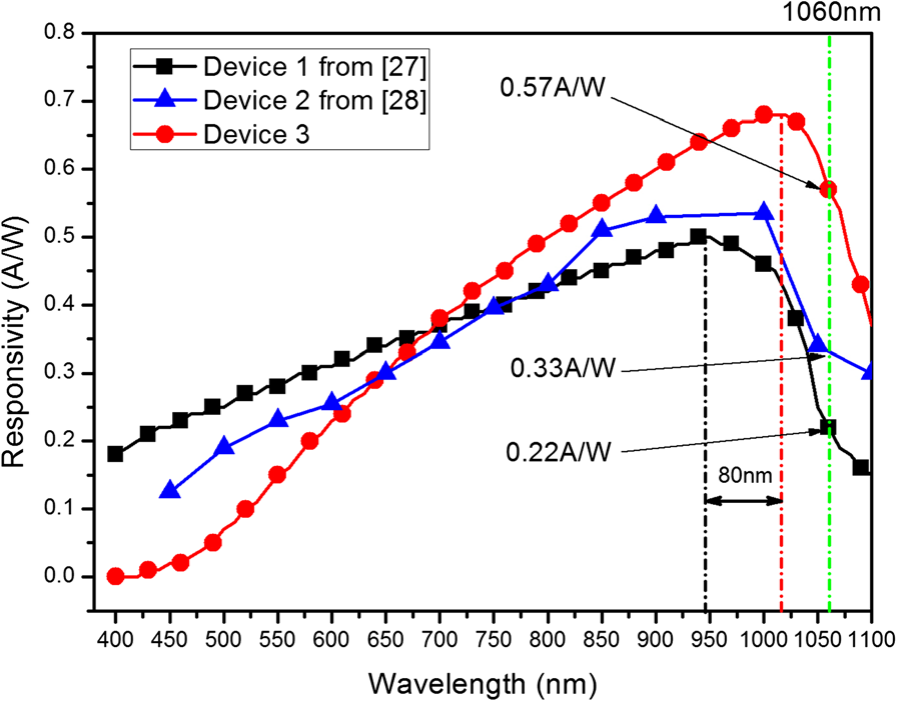
Responsivities of three different detectors: device 1 from ref. [27], device 2 from ref. [28], and device 3 based on the results of present paper

Figure 6 gives the comparison of detector responsivity from three different groups. It is hereby declared that the responsivity of device 1 (S1336-44BK, a commercial Si-PIN detector) is re-plotted based on the public Website of Hamamatsu Photonics Company [27], and the responsivity of Device 2 (Si-PIN detector) with black silicon formed on the back surface is also re-plotted based on the reference [28]. The responsivity of device 3 is obtained on our newly fabricated Si-PIN detector with black silicon formed on the front surface. It can be clearly seen that device 3 performs a substantial increase in responsivity, particularly at near-infrared wavelengths, i.e., 0.57 A/W at 1060 nm and 0.37 A/W at 1100 nm, respectively.

It is well known that the traditional Si-PIN photoelectronic detector is less sensitive to light illumination in the near-infrared band (beyond 1000 nm). The Si-PIN photoelectronic detector based on black silicon, however, shows a high responsivity in the near-infrared wavelength. It is considered that when light is shone onto the photosensitive surface, it repeatedly bounces back and forth between the nanocone arrays with more light trapped. Therefore, the reduced reflectance with light trapping increases the sensitivity of silicon to near-infrared wavelength and makes it viable for use in near-infrared detection. It can be seen from Fig. 6 that although the Si-PIN detector with black silicon formed on the front surface (device 3) shows a relatively low responsivity in visible spectrum, the response spectrum of it gives an even higher responsivity than that of black formed on the back surface (device 2) [28] with about 80-nm red shift of peak responsivity, when the incident light wavelength is greater than 650 nm.

The main reason for such a distinction is that the device structure of these two detectors (device 2 and device 3) is different. For device 3, the black silicon layer is directly set as the photosensitive surface, and when the device works properly, the visible light is mainly absorbed by the N^+^ layer and so the generated carriers begin to recombine inside the N^+^ layer at the same time. It is very difficult for the generated carriers to be collected by the P layer to output photocurrent through electrode. That is why there is only a relatively low visible light response in the measured responsivity curve. Nevertheless, the near-infrared light is able of penetrating N^+^ layer and absorbed by N layer, and then a large number of generated carriers are able to be collected by the P layer under the action of reverse bias. As a result, there will be a countable photocurrent output and the device represents a substantial responsivity increase in the near-infrared wavelength. Another reason might be the scattering effect caused by the specific nanostructures presented in this paper on the surface of silicon substrate. This nanometer effect reduces the difference between direct band gap width and indirect band gap width of the nanostructured black silicon, which means that the band structure of the surface silicon will transform from primary indirect band gap structure into quasi-direct band gap one, thus increasing the absorption coefficient of near-infrared light within the band gap and causing the red shift of absorption wavelength. Therefore, as shown in Fig. 6, the Si-PIN detector with black silicon formed on the front surface (device 3) shows a higher responsivity in the wavelength range of 650 to 1100 nm with a red shift of peak responsivity, compared with the commercial Si-PIN detector (device 1).

Realizing the high light absorptance and broad response spectrum of black silicon is extremely important not only for the fundamental studies of novel performance of silicon material in optical engineering but also for its likeable applications in photoelectronic detectors. Our present study might provide a feasible strategy for these applications, but there are still a lot of aspects should be improved. For example, better fabrication processes of nanostructured black silicon and other kinds of micro/nanostructured black silicon, which could precisely control the morphologies of the structured silicon surfaces at a low cost, should be explored. Furthermore, some other novel device structures of photoelectronic detector based on black silicon should be designed in order to realize a better device performance. Therefore, much more efforts will be focused on these issues in the near future.

## Conclusions

In summary, nanostructured black silicon materials are fabricated by metal-assisted chemical etching, and the nanocone arrays on the silicon surface have diameter and length of 100 nm and 2.5 μm, respectively. Greatly enhanced light absorptance of black silicon has been observed in a wide wavelength range of 400 to 2500 nm, and the maximum absorptance reaches 95 %. This enhancement is explained by reduced reflectance, light-trapping effect, and scattering effect caused by the specific nanostructures on the surface of silicon substrate. A novel Si-PIN photoelectronic detector with black silicon formed on the front surface has been fabricated. The comparison of device responsivities has been made with other two devices, one of which is S1336-44BK from the Website of Hamamatsu Photonics Company [27] and the other is a photoelectronic detector with black silicon formed on the front surface from reference [28]. It is concluded that our Si-PIN photoelectronic detector with nanostructured black silicon formed on the front surface has a substantial increase in responsivity with about 80 nm red shift of peak responsivity, particularly in the near-infrared wavelengths, rising to 0.57 A/W at 1060 nm and 0.37 A/W at 1100 nm, respectively.
